# Supplementary material for: Antioxidant, Anti-tyrosinase, Anti-α-amylase, and Cytotoxic Potentials of the Invasive Weed Andropogon virginicus
Source: Plants (Basel). 2020 Dec 31;10(1):69. doi: 10.3390/plants10010069 (PMC7824498; doi:10.3390/plants10010069)
Supplement: Supplementary file 1 [file plants-10-00069-s001.pdf]

Supplementary Materials:

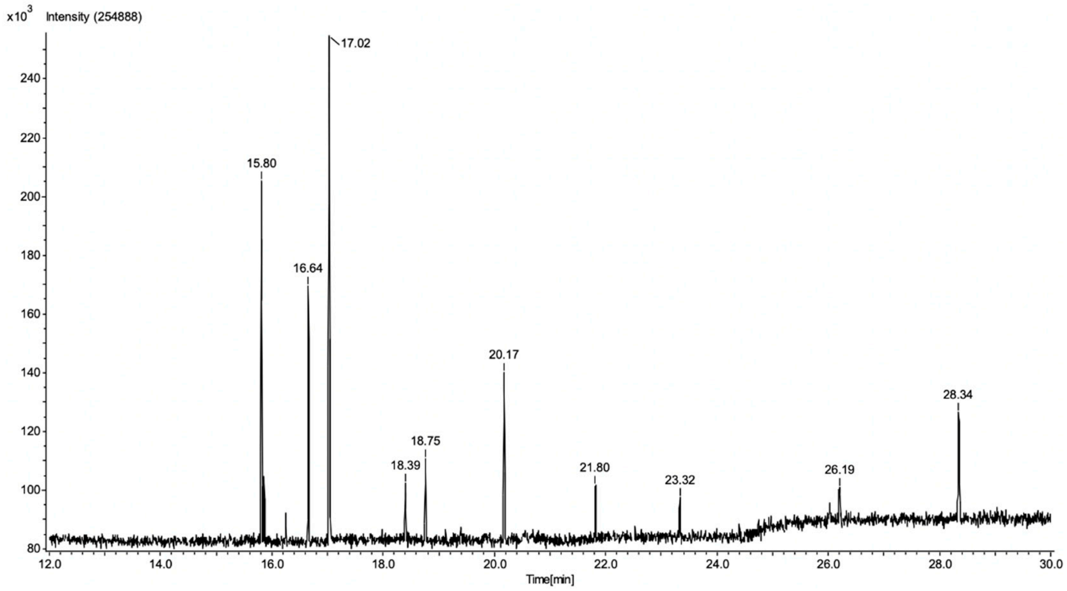

Figure S1. GC-MS chromatogram of H-Anvi extract

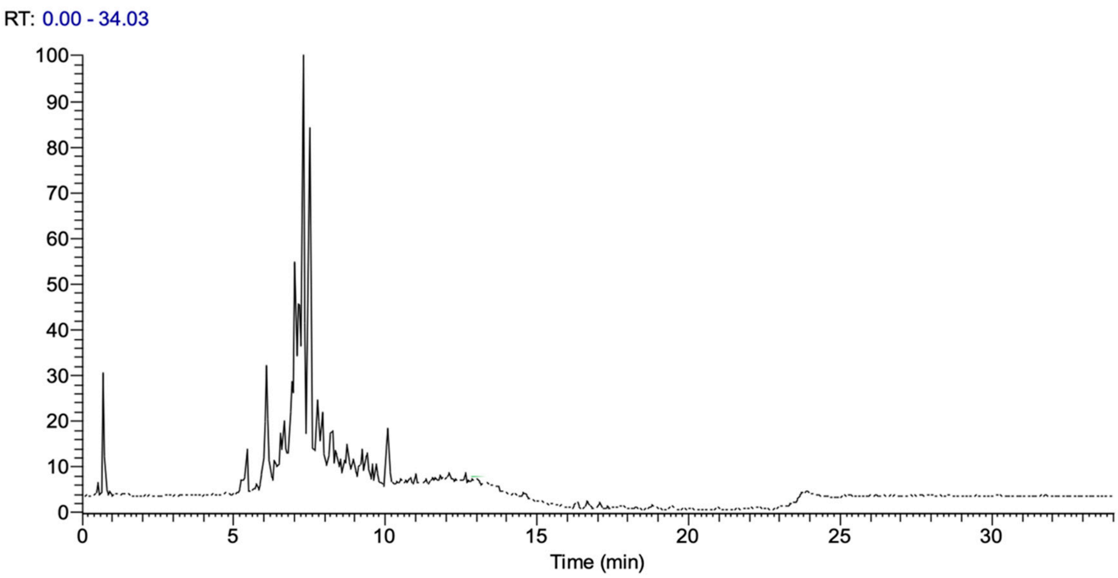

Figure S2. HPLC chromatogram of E-Anvi extract
